# Supplementary material for: Age-Related Changes in Serum N-Glycome in Men and Women—Clusters Associated with Comorbidity
Source: Biomolecules. 2023 Dec 22;14(1):17. doi: 10.3390/biom14010017 (PMC10813383; doi:10.3390/biom14010017)
Supplement: Supplementary file 1 [file biomolecules-14-00017-s001.zip › biomolecules-2748975-supplementary.pdf]

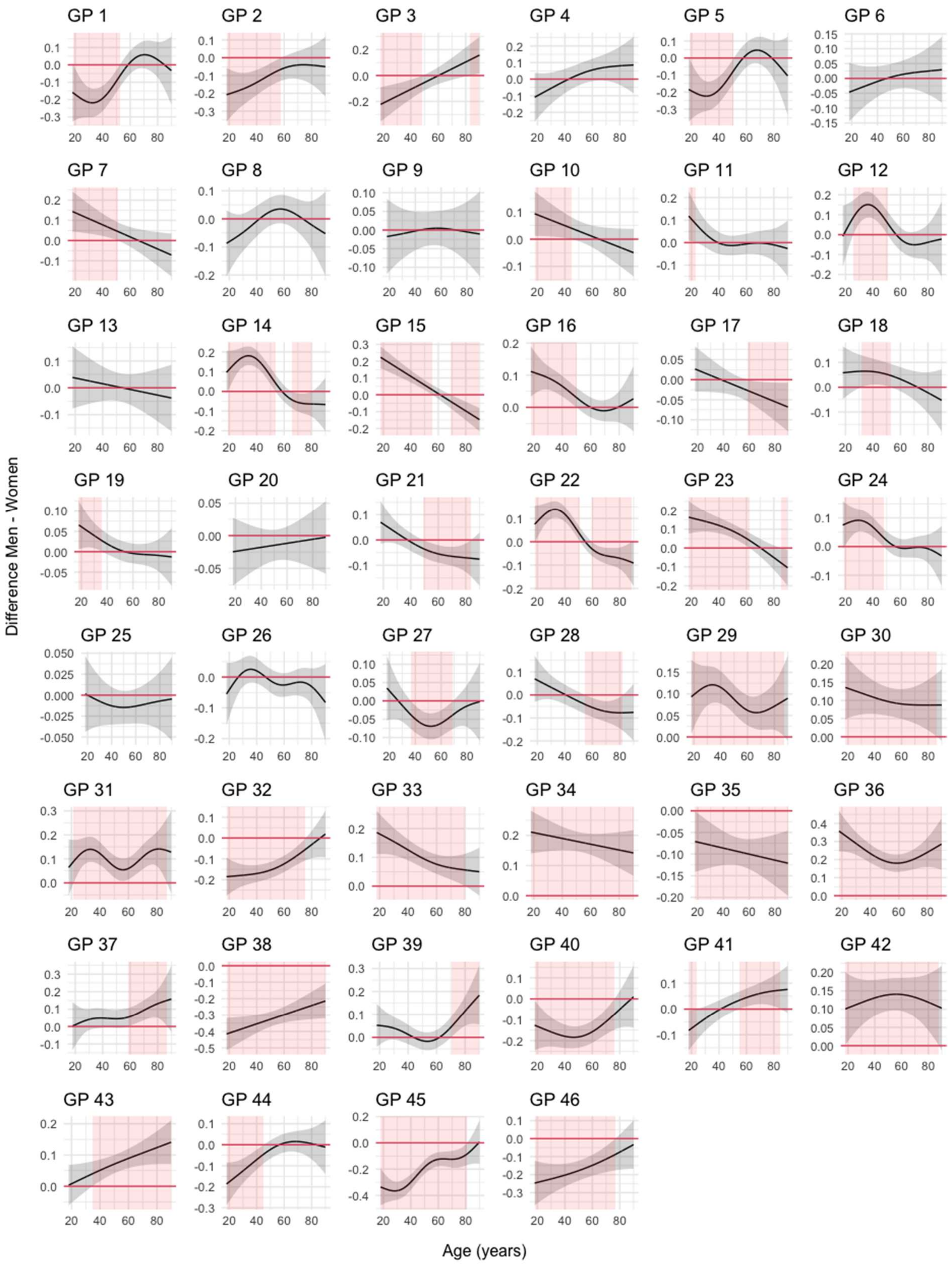

Figure S1. Glycan peak (GP) abundance in relation to age and sex. For each GP, the difference between men and women is represented after transforming the data into ilr (isometric log-ratios), with its 95% confidence interval. The segments shaded in pink correspond to the ages with a statistically significant difference ($P<0.05$) between men and women. The curves were obtained using generalized additive regression models with a predictor factor per curve, estimating a smooth effect of age (using spline functions) for men and women. The adjustments were obtained on the ilr scale.

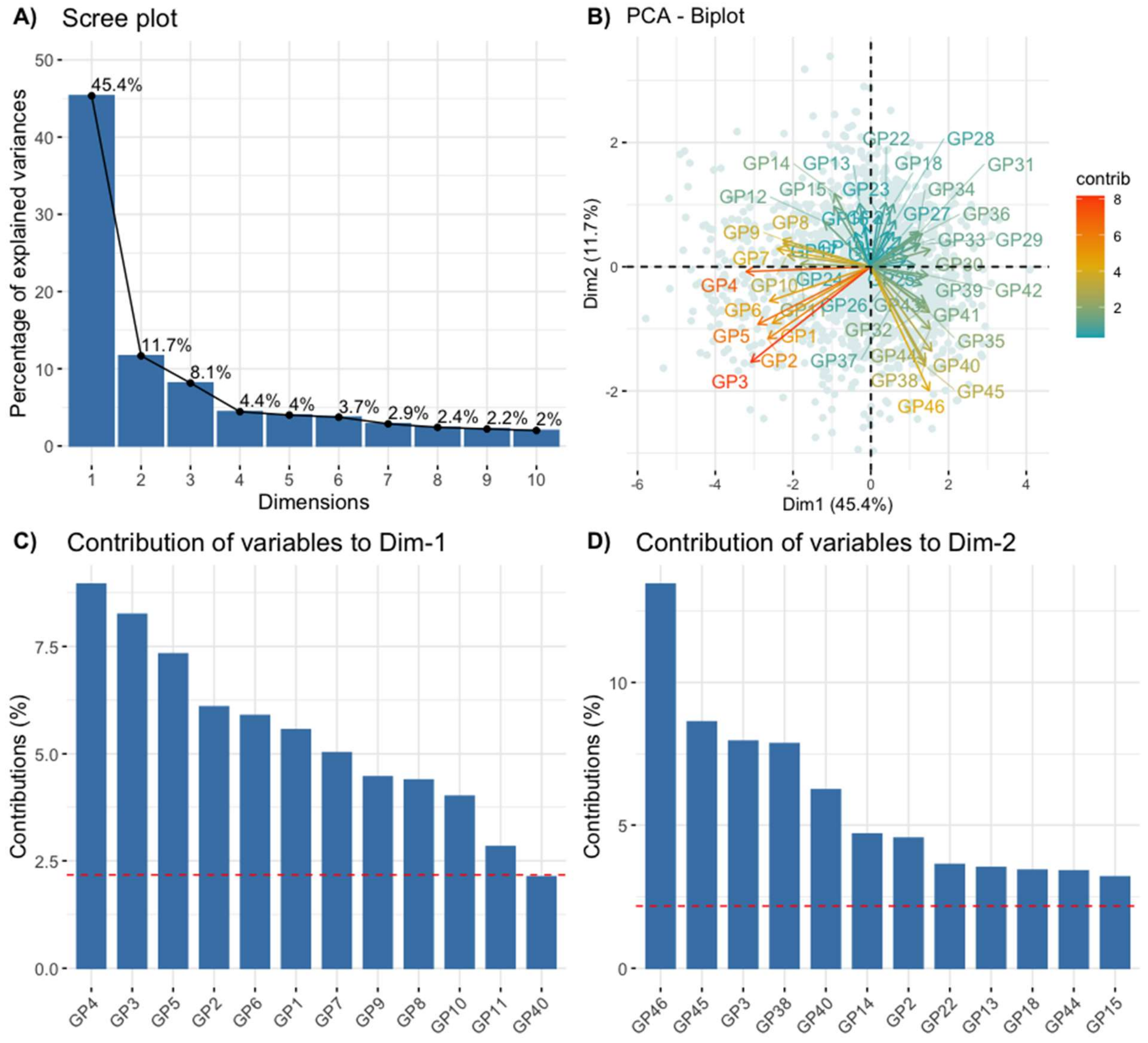

Figure S2. Principal component analysis (PCA) of the 46 glycan peaks (GPs). (A) PCA scree plot. Dimensions 1, 2 and 3 explain more than 60% of the variance of the original variables. Dimensions 1 and 2 alone explain more than 50%. (B) Biplot of the PCA that represents the two main dimensions (Dim) (1 and 2), with the representation of the vectors of each GP. (C) and (D) Contribution of the variables (GPs) to dimensions 1 and 2. The greatest contribution to the first dimension is provided by simple N-glycans (GP1 to GP11; mono- or biantennary, non-sialylated and agalactosylated or monogalactosylated).

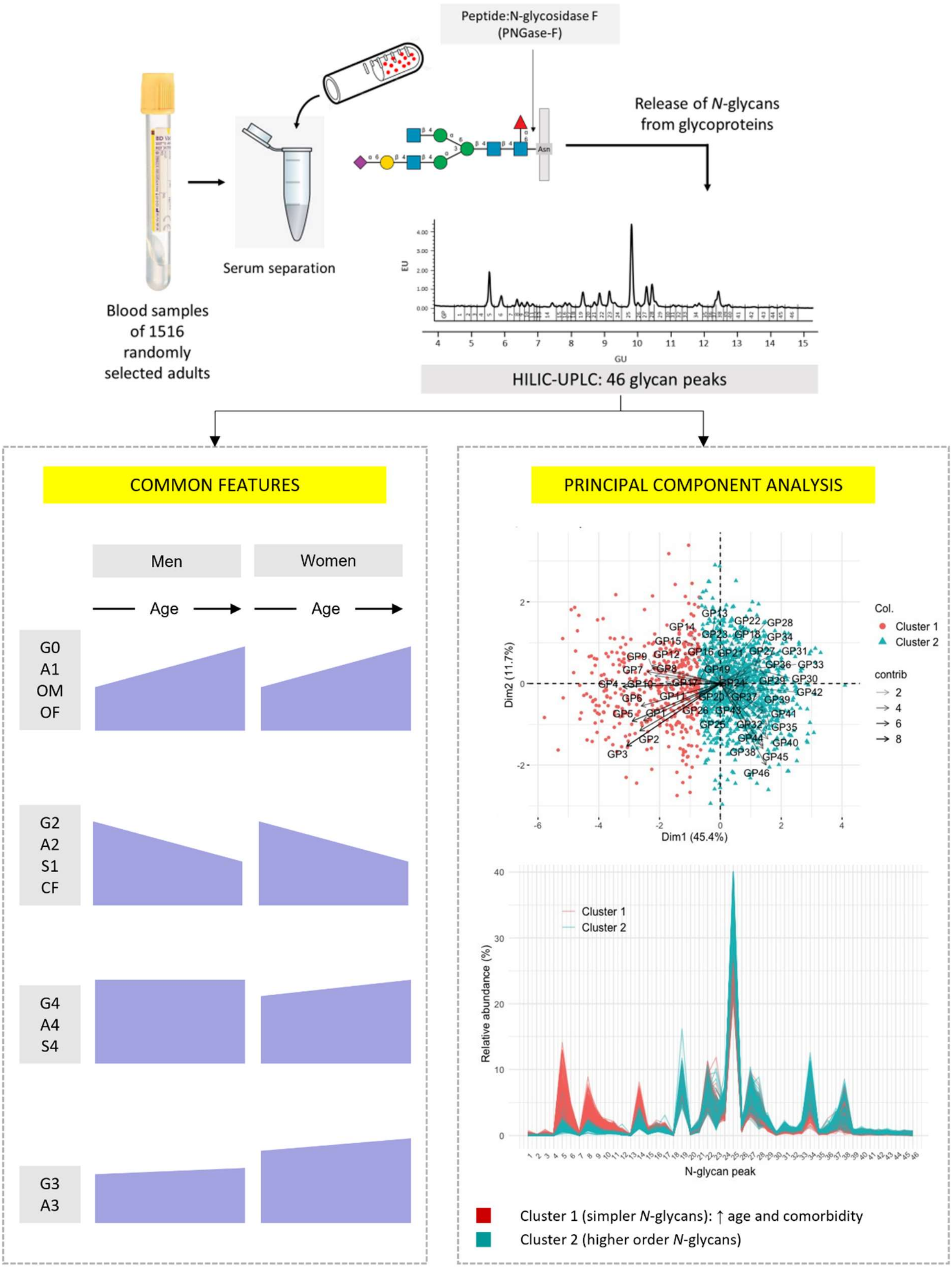

Figure S3. Summary of the main study findings. G0, agalactosylated; G2, digalactosylated; G3, trigalactosylated; G4, tetragalactosylated; A1, monoantennary; A2, biantennary; A3, triantennary; A4, tetraantennary; S1, monosialylated; S4 tretrasialylated; CF, core fucosylation; OF, outer arm fucosylation; OM, oligommannose *N*-glycans.

Table S1. The *N*-glycan composition of the 46 peaks in control serum samples

Peak	Name	Major structure	Peak	Name	Major structure	Peak	Name	Major structure
**GP1**	A1		**GP18**	FA2[3]BG1S[3]1		**GP35**	A3BG3S[3,3,3]3	
**GP2**	FA1		**GP19**	A2G2S[6]1		**GP36**	FA3G3S[3,6,6]3	
**GP3**	A1[6]G1		**GP20**	A2BG2S[6]1		**GP37**	A3F1G3S[3,3,3]3	
**GP4**	A2B		**GP21**	M5A1G1S[3]1		**GP38**	A4G4S[3,3,3]3	
				A2G2S[3]1				
**GP5**	FA2		**GP22**	FA2G2S[6]1		**GP39**	A4G4S[3,3,6]3	
**GP6**	M5		**GP23**	FA2BG2S[3]1			A4G4S[3,6,6]3	
**GP7**	A2[6]BG1							
**GP8**	FA2[6]G1		**GP24**	A2G2S[3,6]2		**GP40**	A4F1G3S[3,3,3]3	
**GP9**	FA2[3]G1		**GP25**	A2G2S[3,6]2			A4F1G3S[3,3,6]3	
**GP10**	FA2[6]BG1		**GP26**	A2BG2S[3,6]2			A4F1G3S[3,6,6]3	
**GP11**	M6 D3		**GP27**	FA2G2S[3,6]2		**GP41**	A4G4S[3,3,3,3]4	
**GP12**	A1[3]G1S[3]1		**GP28**	FA2BG2S[6,6]2		**GP42**	A4G4S[3,3,3,6]4	
	A2G2		**GP29**	A3G3S[3,6]2		**GP43**	A4G4S[3,3,3,6]4	
**GP13**	A2BG2		**GP30**	A4G4S[3]1		**GP44**	A4G4S[3,3,3,3]4	
**GP14**	FA2G2		**GP31**	A3G3S[3,3]2		**GP45**	A4F1G4S[3,3,3,6]4	
**GP15**	FA2BG2		**GP32**	A3G3S[3,3,3]3		**GP46**	A4F3G4S[3,3,3,3]4	
**GP16**	A2[3]BG1S[3]1		**GP33**	A3G3S[3,3,6]3		Only main structures are shown in the table.		
**GP17**	FA2[3]G1S[6]1		**GP34**	A3G3S[3,3,6]3				

▲ Fucose ● Galactose ● Mannose ■ *N*-acetylglucosamine ◆ *N*-acetylneuraminic acid

Table S2. Serum *N*-glycan peak abundance in men and women

**Glycan peak** (%)	**Total sample** (n=1516)	**Women** (n=838)	**Men** (n=678)	***p*-value**
GP1	0.12 [0.08, 0.17]	0.11 [0.08, 0.16]	0.12 [0.09, 0.17]	0.017
GP2	0.04 [0.03, 0.06]	0.04 [0.03, 0.06]	0.04 [0.03, 0.06]	<0.001
GP3	0.08 [0.05, 0.13]	0.08 [0.05, 0.13]	0.08 [0.05, 0.13]	0.503
GP4	0.07 [0.05, 0.10]	0.07 [0.05, 0.10]	0.07 [0.05, 0.10]	0.503
GP5	2.30 [1.63, 3.36]	2.24 [1.58, 3.36]	2.39 [1.72, 3.33]	0.029
GP6	1.03 [0.82, 1.41]	1.03 [0.82, 1.40]	1.02 [0.81, 1.42]	0.619
GP7	0.10 [0.07, 0.13]	0.10 [0.07, 0.13]	0.09 [0.07, 0.13]	0.052
GP8	1.91 [1.53, 2.51]	1.91 [1.55, 2.50]	1.92 [1.49, 2.53]	0.946
GP9	1.05 [0.82, 1.37]	1.05 [0.82, 1.35]	1.04 [0.81, 1.39]	0.909
GP10	0.67 [0.54, 0.87]	0.68 [0.55, 0.86]	0.66 [0.52, 0.89]	0.078
GP11	0.58 [0.48, 0.71]	0.58 [0.48, 0.71]	0.58 [0.47, 0.71]	0.574
GP12	0.32 [0.24, 0.41]	0.32 [0.24, 0.42]	0.31 [0.24, 0.41]	0.091
GP13	0.08 [0.06, 0.10]	0.08 [0.06, 0.10]	0.08 [0.06, 0.10]	0.646
GP14	2.74 [2.23, 3.41]	2.78 [2.25, 3.48]	2.68 [2.21, 3.29]	0.037
GP15	0.50 [0.40, 0.63]	0.51 [0.41, 0.64]	0.49 [0.40, 0.61]	0.017
GP16	1.03 [0.90, 1.19]	1.05 [0.91, 1.21]	1.01 [0.88, 1.16]	0.002
GP17	1.05 [0.88, 1.21]	1.04 [0.88, 1.20]	1.07 [0.88, 1.23]	0.104
GP18	0.18 [0.14, 0.22]	0.18 [0.14, 0.22]	0.18 [0.13, 0.21]	0.054
GP19	7.52 [6.91, 8.15]	7.56 [6.93, 8.17]	7.46 [6.88, 8.13]	0.664
GP20	0.65 [0.58, 0.71]	0.65 [0.58, 0.71]	0.65 [0.58, 0.71]	0.579
GP21	1.29 [1.12, 1.46]	1.27 [1.10, 1.43]	1.30 [1.13, 1.49]	0.024
GP22	5.97 [5.24, 6.96]	5.97 [5.20, 7.17]	5.97 [5.26, 6.83]	0.336
GP23	2.75 [2.27, 3.45]	2.85 [2.34, 3.57]	2.66 [2.18, 3.31]	<0.001
GP24	4.48 [3.99, 4.97]	4.50 [4.03, 5.00]	4.42 [3.95, 4.93]	0.066
GP25	31.52 [29.24, 33.59]	31.31 [29.20, 33.23]	31.76 [29.27, 33.83]	0.244
GP26	1.41 [1.23, 1.60]	1.42 [1.23, 1.59]	1.40 [1.24, 1.60]	0.946
GP27	5.40 [4.65, 6.15]	5.29 [4.61, 5.96]	5.53 [4.74, 6.35]	0.001
GP28	3.16 [2.67, 3.77]	3.12 [2.66, 3.67]	3.20 [2.71, 3.87]	0.037
GP29	1.81 [1.54, 2.07]	1.88 [1.63, 2.10]	1.71 [1.45, 1.99]	<0.001
GP30	0.30 [0.24, 0.36]	0.31 [0.25, 0.37]	0.28 [0.23, 0.35]	<0.001
GP31	1.06 [0.89, 1.23]	1.11 [0.94, 1.27]	0.98 [0.84, 1.16]	<0.001
GP32	0.62 [0.51, 0.74]	0.59 [0.48, 0.70]	0.66 [0.55, 0.78]	<0.001
GP33	0.96 [0.81, 1.12]	0.98 [0.85, 1.15]	0.91 [0.76, 1.08]	<0.001
GP34	6.11 [5.02, 7.29]	6.54 [5.45, 7.58]	5.49 [4.45, 6.65]	<0.001
GP35	0.44 [0.36, 0.54]	0.42 [0.35, 0.52]	0.47 [0.38, 0.56]	<0.001
GP36	0.57 [0.46, 0.72]	0.63 [0.51, 0.78]	0.51 [0.41, 0.62]	<0.001
GP37	1.76 [1.44, 2.15]	1.81 [1.49, 2.19]	1.69 [1.39, 2.05]	<0.001
GP38	3.24 [2.32, 4.21]	2.73 [1.97, 3.72]	3.79 [2.94, 4.79]	<0.001
GP39	0.45 [0.38, 0.54]	0.46 [0.39, 0.55]	0.45 [0.38, 0.52]	0.054
GP40	0.41 [0.32, 0.53]	0.39 [0.30, 0.50]	0.44 [0.34, 0.57]	<0.001
GP41	0.45 [0.38, 0.53]	0.46 [0.38, 0.54]	0.45 [0.38, 0.52]	0.263
GP42	0.28 [0.22, 0.36]	0.30 [0.24, 0.38]	0.27 [0.21, 0.34]	<0.001
GP43	0.42 [0.35, 0.49]	0.43 [0.37, 0.51]	0.40 [0.34, 0.47]	<0.001
GP44	0.22 [0.18, 0.27]	0.22 [0.17, 0.27]	0.23 [0.18, 0.28]	0.034
GP45	0.25 [0.19, 0.32]	0.23 [0.17, 0.29]	0.28 [0.22, 0.34]	<0.001
GP46	0.16 [0.12, 0.22]	0.15 [0.11, 0.20]	0.18 [0.13, 0.24]	<0.001

Data are medians and interquartile ranges (in brackets). The raw data are represented (in percentage of abundance), although for statistical comparisons they were previously transformed into isometric log-ratios (ilr). P values were obtained with the Mann-Whitney test with Benjamini correction. The GPs highlighted in green reflect a significantly greater abundance in men. The GPs highlighted in red-orange reflect a significantly higher abundance in women. GP, glycan peak.

Table S3. Serum *N*-glycan trait abundance in men and women

**Glycan trait** (%)	**Total sample** (n=1516)	**Women** (n=838)	**Men** (n=678)	**P-value**
G0	3.19 [2.40, 4.53]	3.14 [2.37, 4.50]	3.25 [2.47, 4.55]	0.262
G1	6.95 [6.05, 8.26]	6.97 [6.12, 8.10]	6.95 [6.01, 8.39]	0.088
G2	67.98 [65.43, 69.98]	67.79 [65.20, 69.91]	68.22 [65.75, 70.05]	0.112
G3	13.48 [11.54, 15.41]	14.14 [12.36, 15.89]	12.58 [10.70, 14.47]	<0.001
G4	6.22 [5.09, 7.58]	5.84 [4.72, 6.95]	6.78 [5.68, 8.17]	<0.001
A1	1.08 [0.95, 1.22]	1.07 [0.95, 1.21]	1.09 [0.96, 1.24]	0.878
A2	77.76 [75.70, 79.82]	77.59 [75.59, 79.58]	78.02 [75.87, 79.94]	0.033
A3	13.48 [11.54, 15.41]	14.14 [12.36, 15.89]	12.58 [10.70, 14.47]	<0.001
A4	6.22 [5.09, 7.58]	5.84 [4.72, 6.95]	6.78 [5.68, 8.17]	<0.001
S0	11.83 [9.82, 14.69]	11.86 [9.95, 14.58]	11.64 [9.64, 14.89]	0.461
S1	21.15 [19.48, 22.85]	21.25 [19.62, 23.02]	20.99 [19.27, 22.55]	0.134
S2	49.50 [47.16, 51.29]	49.28 [47.09, 51.02]	49.81 [47.49, 51.79]	0.024
S3	14.87 [13.06, 16.59]	14.94 [13.29, 16.65]	14.75 [12.87, 16.49]	0.461
S4	1.82 [1.50, 2.13]	1.82 [1.49, 2.13]	1.82 [1.54, 2.14]	0.461
CF	30.44 [27.23, 33.93]	30.40 [27.27, 33.77]	30.47 [27.08, 34.19]	1.000
OF	2.67 [2.28, 3.12]	2.67 [2.28, 3.14]	2.66 [2.28, 3.11]	1.000
OM	1.10 [0.91, 1.40]	1.09 [0.92, 1.40]	1.10 [0.91, 1.40]	1.000

Data are medians and interquartile ranges (in brackets). The raw data are represented (in percentage of abundance), although for statistical comparisons they were previously transformed into isometric log-ratios (ilr). The fucosylation and oligomannose traits were treated as random variables with restricted domain (0,1). P-values were obtained with the Mann-Whitney test with Benjamini correction. The traits highlighted in green reflect a significantly greater abundance in men. The traits highlighted in red-orange reflect a significantly higher abundance in women. G, galactosylation (G0 a-, G1 mono, G2 di-, G3 tri-, G4 tetra-galactosylated). A, antennae (branching: A1 mono-, A2 di-, A3 tri-, A4 tetra-antennary). S, sialylation (S0 a-, S1 mono-, S2 di-, S3 tri-, S4 tetra-sialylated). F, fucosylation (CF, core-fucosylation; OF, outer arm fucosylation). OM, oligomannose.

Table S4. Serum *N*-glycan trait abundance in men and women according to age strata

**Glycan trait** (%)		**18-40 years**	**41-60 years**	**61-90 years**	**P-value**
G0	Women	2.45 [1.88,3.25]	2.99 [2.34,4.17]	3.98 [3.04,5.47]	<0.001
	Men	2.90 [2.22,4.08]	3.06 [2.41,4.15]	3.90 [2.95,5.30]	<0.001
G1	Women	6.95 [6.05,7.84]	7.04 [6.22,8.10]	6.95 [6.05,8.29]	<0.001
	Men	7.01 [6.09,8.54]	6.72 [5.80,8.27]	7.03 [6.10,8.35]	<0.001
G2	Women	69.52 [67.24,71.24]	67.88 [65.34,70.05]	66.70 [64.19,68.49]	<0.001
	Men	69.53 [66.98,70.98]	67.99 [65.60,69.64]	67.61 [65.52,69.36]	<0.001
G3	Women	13.84 [12.15,15.76]	14.12 [12.46,15.75]	14.25 [12.41,16.13]	<0.001
	Men	12.20 [10.39,13.88]	13.14 [11.19,14.99]	12.63 [10.83,14.48]	0.035
G4	Women	5.48 [4.42,6.57]	5.74 [4.71,6.96]	6.10 [5.04,7.27]	0.945
	Men	6.61 [5.71,7.80]	7.10 [5.79,8.39]	6.69 [5.60,8.02]	0.052
A1	Women	1.00 [0.89,1.14]	1.09 [0.96,1.24]	1.12 [0.99,1.30]	0.006
	Men	1.04 [0.93,1.17]	1.09 [0.97,1.23]	1.17 [1.03,1.35]	<0.001
A2	Women	79.36[77.16,81.48]	78.45 [76.37,80.52]	78.13 [76.18,79.99]	<0.001
	Men	79.76 [77.37,81.75]	78.38 [75.96,80.80]	79.03 [76.86,81.11]	<0.001
A3	Women	13.97 [12.26,15.91]	14.25 [12.67,16.02]	14.45 [12.59,16.32]	0.240
	Men	12.34 [10.56,14.03]	13.23 [11.32,15.17]	12.83 [11.02,14.68]	0.308
A4	Women	5.56 [4.49,6.63]	5.82 [4.76,7.05]	6.17 [5.12,7.35]	0.093
	Men	6.68 [5.78,7.90]	7.16 [5.85,8.47]	6.76 [5.67,8.12]	0.180
S0	Women	11.66 [9.88,13.68]	11.75 [9.82,14.48]	12.10 [10.10,15.07]	0.760
	Men	11.86 [9.92,15.07]	11.17 [9.30,13.99]	12.08 [9.89,15.03]	0.074
S1	Women	22.28 [20.80,24.17]	21.25 [19.75,23.06]	20.40 [18.97,22.08]	<0.001
	Men	21.42 [19.95,22.90]	20.60 [18.99,22.35]	20.66 [19.13,22.48]	0.011
S2	Women	49.00 [46.69,50.56]	49.17 [47.09,50.96]	49.65 [47.14,51.43]	0.196
	Men	49.53 [47.28,51.62]	50.00 [48.05,52.01]	49.58 [47.32,51.75]	0.323
S3	Women	14.55 [12.82,16.27]	15.02 [13.41,16.65]	15.21 [13.64,16.78]	0.433
	Men	14.27 [12.75,16.10]	15.20 [13.38,17.26]	14.69 [12.96,16.21]	0.001
S4	Women	1.73 [1.40,2.01]	1.84 [1.47,2.11]	1.91 [1.57,2.24]	<0.001
	Men	1.78 [1.57,2.13]	1.85 [1.54,2.15]	1.81 [1.49,2.14]	0.786
CF	Women	31.59 [28.82,34.93]	30.44 [27.90,33.55]	29.04 [26.07,33.13]	<0.001
	Men	31.26 [27.85,35.13]	29.56 [26.71,33.87]	30.43 [27.05,33.58]	0.067
OF	Women	2.33 [1.98,2.75]	2.69 [2.33,3.15]	2.85 [2.46,3.31]	<0.001
	Men	2.48 [2.14,2.92]	2.77 [2.39,3.22]	2.72 [2.33,3.16]	<0.001
OM	Women	1.01 [0.84,1.20]	1.07 [0.90,1.34]	1.19 [1.01,1.52]	<0.001
	Men	1.02 [0.83,1.31]	1.05 [0.88,1.31]	1.20 [1.00,1.53]	<0.001

Data are medians and interquartile ranges (in brackets). The raw data are represented (in percentage of abundance), although for statistical comparisons they were previously transformed into isometric log-ratios (ilr). The fucosylation and oligomannose traits were treated as random variables with restricted domain (0,1). P-values were obtained with the Kruskal-Wallis test with Benjamini correction. G, galactosylation (G0 a-, G1 mono, G2 di-, G3 tri-, G4 tetra-galactosylated). A, antennae (branching: A1 mono-, A2 di-, A3 tri-, A4 tetra-antennary). S, sialylation (S0 a-, S1 mono-, S2 di-, S3 tri-, S4 tetra-sialylated). F, fucosylation (CF, core-fucosylation; OF, outer arm fucosylation). OM, oligomannose.

Table S5. Age- and sex-adjusted differences in laboratory determinations between individuals from cluster 1 (n=431) and cluster 2 (n=1085)

	**Age- and sex-adjusted marginal mean difference (cluster 1 – cluster2)**	**P-value**
**Body mass index**, kg/m^2	0.01 (-0.52, 0.55)	0.963
**Serum glucose**, mg/dL	1.26 (-1.12, 3.65)	0.299
**Blood glycated hemoglobin (HbA1c)**, %	-0.02 (-0.10, 0.06)	0.617
**Serum glycated albumin**, %	0.58 (0.31, 0.86)	<0.001
**Serum fructosamine**, µmol/L	14.08 (7.26-20.90)	<0.001
**Serum HDL-cholesterol**, mg/dL	2.82 (1.04, 4.60)	0.002
**Serum LDL-cholesterol**, mg/dL	-0.83 (-4.36, 2.70)	0.646
**Erythrocyte sedimentation rate (ESR)**, mm/h	0.48 (-0.70, 1.67)	0.423
**Serum C-reactive protein**, mg/dL	-0.07 (-0.15, 0.00)	0.061
**Serum TNF-alpha**, pg/mL	0.31 (-0.01, 0.64)	0.061
**Serum interleukin-8 (IL-8)**, pg/mL	-1.22 (-5.27, 2.82)	0.554
**Serum interleukin-6 (IL-6)**, pg/mL	-0.07 (-0.92, 0.78)	0.876
**Serum soluble interleukin-2 receptor**, U/mL	0.12 (-17.73, 17.97)	0.989
**Serum aspartate aminotransferase (AST)**, IU/L	0.96 (0.07, 1.85)	0.034
**Gamma-glutamyl transferase (GGT)**, IU/L	5.34 (0.79, 9.90)	0.022
**Serum triiodothyronine (T3)**, pg/mL	-0.09 (-0.15, -0.03)	0.003
**Glomerular filtration rate**, mL/min/1.7 m^2	-3.26 (-5.68, -0.84)	0.008

Data are marginal mean differences with 95% confidence intervals (in parentheses). LDL, low-density lipoprotein; HDL, high-density lipoprotein; TNF, tumor necrosis factor.

Supplementary Methods S1 - Serum *N*-glycan analyses

N-glycans were released from 5 µL of serum samples using a modified high-throughput automated method [*Stöckmann et al., 2015*]. Briefly, the samples were denaturated with dithiothreitol, alkylated with iodoacetamide, and *N*-glycans were released from the protein backbone enzymatically via PNGase F (NEB Recombinant, code P0709L, 10 µL per well, 5000 U in 1 M ammonium bicarbonate, pH 8.0, room temperature). Glycans were immobilized on solid supports, and excess reagents were removed by centrifuge filtration. Glycans were released from the solid supports and labelled with the fluorophore 2-aminobenzamide (2-AB) as described [*Stöckmann et al., 2015*]. Glycans were cleaned up using 96-well chemically inert filter plate (Millipore Solvinert, hydrophobic polytetrafluoroethylene membrane, 0.45 µm pore size) using HyperSep Diol SPE cartridges (Thermo Scientific).

For hydrophilic interaction chromatography (HILIC) ultra-performance liquid chromatography (UPLC), fluorescently labelled *N*-glycans were separated on a Waters Acquity H-Class UPLC instrument (Milford, MA, USA) consisting of a quaternary solvent manager, sample manager-FTN, column manager and a FLR fluorescence detector set with excitation and emission wavelengths of 245 and 395 nm, respectively. The instrument was under the control of Empower 3 software, build 3471 (Waters, Milford, MA, USA). Labelled *N*-glycans were separated on a Waters Ethylene Bridged Hybrid BEH Glycan chromatography column, 150 × 2.1 mm i.d., 1.7 µm BEH particles, with 50 mM ammonium formate, pH 4.4, as solvent A and MeCN as solvent B. The column was fitted with an ACQUITY in-line 0.2 µm filter. Separation method used linear gradient of 70–30% acetonitrile (v/v) at flow rate of 0.56 mL/min in a 30 min analytical run. Samples were maintained at 4 °C before injection. An injection volume of 25 µL sample prepared in 70% v/v MeCN was used throughout. Samples were maintained at 5 °C prior to injection and the separation temperature was 40 °C. The system was calibrated using an external standard of hydrolyzed and 2-AB labelled glucose oligomers to create a dextran ladder, as described previously [*Royle et al., 2008*]. A fifth-order polynomial distribution curve was fitted to the dextran ladder to assign glucose unit (GU) values from retention times (using Empower software from Waters). The chromatograms were all separated in the same manner into 46 peaks according to *Saldova et al.* (2014), and the amount of glycans in each peak was expressed as the percentage of total integrated area. Glycan structures were annotated using the Symbol Nomenclature for Glycans (SNFG) and the DrawGlycan-SNFG software [*Cheng et al., 2016; Neelamegham et al., 2019*] with the assist of GlycoStore.org [*Zhao et al., 2018*].

Cheng, K.; Zhou, Y.; Neelamegham, S. DrawGlycan-SNFG: a robust tool to render glycans and glycopeptides with fragmentation information. *Glycobiology* 2016, 27, 200–205.

Neelamegham, S.; Aoki-Kinoshita, K.; Bolton, E.; Frank, M.; Lisacek, F.; Lütteke, T.; O'Boyle, N.; Packer, N.H.; Stanley, P.; Toukach, P.; Varki, A.; Woods, R.J.; SNFG Discussion Group. Updates to the symbol nomenclature for glycans guidelines. *Glycobiology* 2019, 29, 620–624.

Royle, L; Campbell, M.P.; Radcliffe, C.M.; White, D.M.; Harvey, D.J.; Abrahams, J.L.; Kim, Y.G.; Henry, G.W.; Shadick, N.A.; Weinblatt, M.E.; Lee, D.M.; Rudd, P.M.; Dwek, R.A. HPLC-based analysis of serum N-glycans on a 96-well plate platform with dedicated database software. *Anal. Biochem.* 2008, 376, 1–12.

Saldova, R.; Asadi Shehni, A.; Haakensen, V.D.; Steinfeld, I.; Hilliard, M.; Kifer, I.; Helland, A.; Yakhini, Z.; Børresen-Dale, A.L.; Rudd, P.M. Association of N-glycosylation with breast carcinoma and systemic features using high-resolution quantitative UPLC. *J. Proteome Res.* 2014, 13, 2314–2327.

Stöckmann, H.; O'Flaherty, R.; Adamczyk, B.; Saldova, R.; Rudd, P.M. Automated, high-throughput serum glycoprofiling platform. *Integr. Biol. (Camb)* 2015, 7, 1026–1032.

Zhao, S.; Walsh, I.; Abrahams, JL.; Royle, L.; Nguyen-Khuong, T.; Spencer, D.; Fernandes, D.L.; Packer, N.H.; Rudd, P.M.; Campbell, M.P. GlycoStore: a database of retention properties for glycan analysis. *Bioinformatics* 2018, 34, 3231–3232.
